# First detection of natural infection of *Aedes aegypti* with Zika virus in Brazil and throughout South America

**DOI:** 10.1590/0074-02760160332

**Published:** 2016-10-03

**Authors:** Anielly Ferreira-de-Brito, Ieda P Ribeiro, Rafaella Moraes de Miranda, Rosilainy Surubi Fernandes, Stéphanie Silva Campos, Keli Antunes Barbosa da Silva, Marcia Gonçalves de Castro, Myrna C Bonaldo, Patrícia Brasil, Ricardo Lourenço-de-Oliveira

**Affiliations:** 1 Fundação Oswaldo Cruz, Instituto Oswaldo Cruz, Laboratório de Mosquitos Transmissores de Hematozoários, Rio de Janeiro, RJ, Brasil; 2 Fundação Oswaldo Cruz, Instituto Oswaldo Cruz, Laboratório de Biologia Molecular de Flavivírus, Rio de Janeiro, RJ, Brasil; 3 Instituto Nacional de Infectologia, Laboratório de Pesquisa Clínica em Doenças Febris Agudas, Rio de Janeiro, RJ, Brasil

**Keywords:** Zika, Aedes aegypti, natural infection, Culex quinquefasciatus, Aedes albopictus

## Abstract

Zika virus (ZIKV) has caused a major epidemic in Brazil and several other American countries. ZIKV is an arbovirus whose natural vectors during epidemics have been poorly determined. In this study, 1,683 mosquitoes collected in the vicinity of ZIKV suspected cases in Rio de Janeiro, Brazil, from June 2015 to May 2016 were screened for natural infection by using molecular methods. Three pools of *Aedes aegypti* were found with the ZIKV genome, one of which had only one male. This finding supports the occurrence of vertical and/or venereal transmission of ZIKV in *Ae. aegypti* in nature*.* None of the examined *Ae. albopictus* and *Culex quinquefasciatus* was positive. This is the first report of natural infection by ZIKV in mosquitoes in Brazil and other South American countries. So far, *Ae. aegypti* is the only confirmed vector of ZIKV during the ongoing Pan-American epidemics.

Zika virus (ZIKV) is an arbovirus belonging to the genus *Flavivirus* (family *Flaviviridae*) that originally circulated in enzootic cycles between sylvatic canopy-feeder mosquitoes and non-human primates in Africa (Dick et al. 1952, Weinbren & Williams 1958, Haddow et al. 1964). The virus recently emerged in the Pacific Ocean and subsequently caused a dramatic Pan-American epidemic after its first appearance in Brazil in 2015 (Duffy et al. 2009, Musso et al. 2014, Zanluca et al. 2015, Brasil et al. 2016). Although the virus can be transmitted between humans, transmission by mosquito bite is believed to be the most common way of virus dispersion in epidemic and endemic zones (Hills et al. 2016, Musso & Gubler 2016). *Aedes (Stegomyia) aegypti* has traditionally been considered to be the primary ZIKV vector to humans. It has been confirmed to be competent in transmitting the virus in laboratory assays (Boorman & Porterfield 1956, Chouin-Carneiro et al. 2016). Virus detection in field-caught specimens has been reported, although very infrequently (Marchette et al. 1969, Akoua-Koffi et al. 2001). While natural infections of ZIKV have been found in several hematophagous species in the enzootic cycles in Africa, viral detection in mosquitoes in epidemic urban areas have intriguingly been scarce to non-existent (Haddow et al. 1964, Faye et al. 2013, Diallo et al. 2014, Diagne et al. 2015, Musso & Gubler 2016). Except for finding the ZIKV genome in *Ae. albopictus* and *Ae. aegypti* in Mexico, the virus was not detected in mosquitoes during ZIKV epidemics in the Pacific Ocean and in the American continent (Guerbois et al. 2016, PAHO/WHO 2016). However, detection of infections in wild-caught mosquitoes is imperative for determining the natural vectors. In this study, we report natural infections in mosquitoes during the Zika outbreak in Rio de Janeiro (Brasil et al. 2016), with the goal of determining the local natural ZIKV vectors.

Mosquitoes were caught indoors and around domiciles of suspected Zika cases soon after the emergence of ZIKV in Rio de Janeiro (Brasil et al. 2016), from June 2015 to May 2016. Mosquito captures were performed with backpack aspirators in a sample of households of suspected ZIKV infections as well as in adjacent dwellings. Captures were done during the same week as a medical interview and clinical diagnosis (Brasil et al. 2016). The insects were transported live to the laboratory, where they were sexed, identified to species, and visually screened according to feeding status on cold plates. Males and visually non-blood-fed females were pooled according to species, gender, and collection site, and were stored at a low temperature (N_2_ or -80ºC) until tested. The abdomen of 57% of the females was detached prior to examination if there were doubt about the efficacy of visual screening according to feeding status. The number of mosquitoes by pool varied from one to 10, consistent with the number of captured specimens at each site and day of collection. Mosquito pools were ground in Leibovitz L15 medium (Invitrogen) supplemented with 20% foetal bovine serum and centrifuged at 10,000 ×*g* for 5 min at 4ºC. Mosquito homogenates were screened through real-time quantitative polymerase chain reaction (RT-qPCR) using the SuperScript III Platinum one-step RT-qPCR (Invitrogen) in QuantStudio 6 Flex Real-Time PCR System (Applied Biosystems). For each reaction, we used 600 nM forward primer (5′-CTTGGAGTGCTTGTGATT-3′, genome position 3451-3468), 600 nM reverse primer (5′-CTCCTCCAGTGTTCATTT-3′, genome position 3637-3620), and 800 nM probe (5′FAM- AGAAGAGAATGACCACAAAGATCA-3′TAMRA, genome position 3494-3517). The reverse transcription was performed at 45ºC for 15 min. The sequences of this primer set were kindly provided by Isabelle Lepark-Goffart (French National Reference Centre for Arboviruses, IRBA, Marseille, France). The qPCR conditions were 95ºC for 2 min, followed by 40 amplification cycles of 95ºC for 15 s, 58ºC for 5 s, and 60ºC for 30 s. For each run, numbers of ZIKV genomic RNA were calculated by absolute quantitation using a standard curve (Bonaldo et al. 2016).

To confirm ZIKV infection, the homogenate of the mosquito pool, positive when tested by RT-qPCR, was submitted to a RT-PCR. Initially, the viral RNA was reverse transcribed with the Superscript IV First-Strand Synthesis System (Invitrogen) and random hexamers primers, according to the manufacturer’s recommendations. The reaction was carried out at 23ºC for 10 min, 55ºC for 10 min, and 80ºC for 10 min. Then, the viral single-stranded cDNA was amplified by conventional PCR using GoTaq Green Master Mix (Promega) according to the manufacturer’s recommendations. We performed two different analyses to detect viral RNA sequences in mosquito samples. In the first, employing the primers 5′-GCTACTGGATTGAGAGTGAGAAG-3′ (genome position: 3085 to 3107), and 5′-CTCAGAGATGGTCCTCTTGTTC-3′ (genome position 3364 to 3385), it was possible to detect an expected 300 bp band. In the second analysis, we utilised a semi-nested PCR protocol. The primers were 5′-AGAGAATCTGGAGTACCGGATAA-3′ (1373-1395) and 5′-GTATGACACGCCCTTCAATCT-3′ (1872-1892) for the first step of amplification generating a 520 bp amplicon and 5′-GGCAAACTGTCGTGGTTCTA-3′ (1732-1751) for the second-step PCR raising a 161 bp fragment. The thermocycling program set up in a Veriti 96 Well thermocycler (Applied Biosystem) was one cycle of 95ºC for 5 min, 40 cycles of 95ºC for 40 s, 50ºC for 40 s, 72ºC for 30 s, one cycle of 72ºC for 10 min, and a hold of 4ºC for the first primer set. The next primer set was performed at one cycle of 95ºC for 5 min, 40 cycles of 95ºC for 40 s, 50ºC for 40 s, 72ºC for 35 s, one cycle of 72ºC for 10 min, and a hold of 4ºC. Finally, 10 µL of amplified products were detected by electrophoresis on a 2% agarose gel, visualised by ethidium bromide staining UV. Additionally, PCR reaction products were purified using a QIAquick PCR purification Kit according to the manufacturer’s recommendations. Samples were then sequenced in both directions using the primers utilised in the PCR protocol on an ABI Prism 3730 Genetic Analyzer with BigDye Terminator, version 3.1.

A total of 1,683 mosquitoes (720 females and 963 males), belonging to three species, were screened for ZIKV infection, as follows: 550 *Ae. aegypti* (315 females and 235 males, grouped in 198 pools), 26 *Ae. albopictus* (20 and 6; 21 pools) and 1,107 *Culex quinquefasciatus* (385 and 722; 249 pools). Three pools of *Ae. aegypti* were positive for ZIKV: P08 and P09 respectively consisted of one female and one male collected in the district of Coelho da Rocha, municipality of São João de Meriti, and P17 had three females collected in the neighbourhood of Realengo, municipality of Rio de Janeiro. The cycle thresholds of positive pools, when screened by RT-qPCR, were 36.68 (P08), 37.78 (P09), and 38.04 (P17). The presence of ZIKV genome in the three pools was confirmed when tested by RT-PCR and semi-nested-PCR using distinct set of primers. The nucleotide sequences of P08 (134 amplicon), P09 (300 and 161 amplicons) and P17 (520 amplicon), covering structural and nonstructural viral genome regions, were determined. By means of Basic Local Alignment Search Tool (BLAST; http://blast.ncbi.nlm.nih.gov/Blast.cgi), the nucleotide sequences (Supplementary data, Figs 1,2) displayed identity with the ZIKV strains of the outbreak occurring in the Americas (e.g., Bonaldo et al. 2016). Viral isolation could not be achieved from these *Ae. aegypti* positive pools because of yeast and bacterium contamination of the inoculated Vero cell monolayers. No other mosquito species tested positive for ZIKV.

This study is the first report of natural infection by ZIKV in mosquitoes in Brazil and other South American countries. Given the relevance of the challenges posed by ZIKV for the Public Health Emergency of International Concern (PHEIC) and the importance of broad sharing of scientific data, the preliminary results of the present study were widely publicised in May 2016 [http://www.sciencemag.org/news/2016/05/top-mosquito-suspect-found-infected-zika (doi:10.1126/science.aaf5743); https://journosdiary.com/2016/05/27/finally-mosquito-species-transmitting-zika-virus-confirmed/; http://www.efe.com/efe/america/sociedad/expertos-brasilenos-confirman-que-aedes-es-el-principal-transmisor-del-zika/20000013-2934094; http://www.theglobeandmail.com/news/world/new-brazilian-research-may-confirm-the-mosquito-source-of-zika-virus/article30118331/].

In the present study, only *Ae. aegypti* mosquitoes were found to be naturally infected. ZIKV was detected in field-collected *Ae. aegypti* in Asia, Africa, and North America, where this mosquito has been identified as ZIKV primary vectors (Marchette et al. 1969, Olson et al. 1981, Akoua-Koffi et al. 2001, Guerbois et al. 2016).

A remarkable finding of the present study was the detection of ZIKV infection in a male *Ae. aegypti* from Rio de Janeiro. As far as we know, this is the first detection of ZIKV infection in a male *Ae. aegypti*. Natural infection by ZIKV in male mosquitoes has only previously been reported in field collected *Aedes furcifer* from Senegal (Diallo et al. 2014). These findings together support the occurrence of vertical and/or venereal transmission of ZIKV in nature, a subject that deserves more investigation in light of its importance to ZIKV transmission dynamics.

Besides *Ae. aegypti*, other *Aedes* (*Stegomyia)* mosquitoes have been shown to be potential natural vectors of ZIKV (Duffy et al. 2009, Ledermann et al. 2014, Musso & Gubler 2016). This is the case of *Ae. albopictus*, which was found naturally infected with the virus in Gabon in 2007 (Grard et al. 2014) and in Mexico in 2016 (PAHO/WHO 2016). In addition, an American population of *Ae. albopictus* (Vero Beach, Florida, USA) was able to transmit ZIKV in the laboratory, although with low efficiency (Chouin-Carneiro et al. 2016). Thus, we cannot rule out the possibility that *Ae. albopictus* may transmit ZIKV in Brazil, and specific control strategies against this mosquitoes should be designed. However, given the very low *Ae. albopictus* capture rates indoors near dwellings with ZIKV cases in Rio de Janeiro, the lack of detection of ZIKV infection in wild specimens reported herein, and the usually low house index reported for *Ae. albopictus* in most Brazilian cities where Zika epidemics have been reported (Carvalho et al. 2014), there is still no sufficient evidence that this mosquito plays any role in ZIKV transmission in Rio de Janeiro, and throughout Brazil.

In contrast, *Ae. aegypti* fulfils all the requirements of a primary ZIKV vector in Rio de Janeiro, and throughout Brazil. It is a very anthropophilic and domestic mosquito with high vectorial capacity to transmit arboviruses (Juliano et al. 2014, Lourenço-de-Oliveira 2015). Together with *Cx. quinquefasciatus,* it is the most frequently occurring indoor man-biting mosquito in urban and suburban Zika epidemic sites in Rio de Janeiro, and throughout Brazil. However, while *Cx. quinquefasciatus* from Rio de Janeiro is not competent to transmit locally circulating ZIKV strains (Fernandes et al. 2016), Brazilian *Ae. aegypti* usually exhibits very high ZIKV transmission efficiency. As expected, vector competence to ZIKV depends on the specific pairing of mosquito populations and virus genotypes. Accordingly, one Brazilian *Ae. aegypti* population challenged with a New Caledonian ZIKV isolate exhibited low vector competence in the laboratory (Choin-Carneiro et al. 2016). But other Brazilian *Ae. aegypti* populations subsequently exposed to four ZIKV isolates from Brazil consistently exhibited high transmission rates and efficiency (Dutra et al. 2016, Fernandes et al. 2016).

Therefore, the present finding of natural infections in *Ae. aegypti* in Brazil, and in Mexico (Guerbois et al. 2016), combined with the competence for ZIKV transmission experimentally determined in Brazilian and other American populations, reinforce *Ae. aegypti* as the primary and the only confirmed vector of ZIKV during the ongoing Pan-American epidemics.
