# Supplementary material for: First detection of natural infection of Aedes aegypti with Zika virus in Brazil and throughout South America
Source: Mem Inst Oswaldo Cruz. 2016 Oct 3;111(10):655–8. doi: 10.1590/0074-02760160332 (PMC5066335; doi:10.1590/0074-02760160332)
Supplement: Supplementary file 1 [file 0074-0276-mioc-0074-02760160332-suppl01.pdf]

```
ZIKV   GCTACTGGATTGAGAGTGAGAAGAATGACACATGGAGGCTGAAGAGGGCCCATCTGATCG
ZP08   GCTACTGGATTGAGAGTGAGAAGAATGACACATGGAGGCTGAAGAGGGCCCATCTGATCG
ZP09     TACTGGATTGAGAGTGAGAAGAATGACACATGGAGGCTGAAGAGGGCCCATCTGATCG
       ************************************************************

ZIKV   AGATGAAAACATGTGAATGGCCAAAGTCCCACACATTGTGGACAGATGGAATAGAAGAGA
ZP08   AGATGAAAACATGTGAATGGCCAAAGTCCCACACATTGTGGACAGATGGAATAGAAGAGA
ZP09   AGATGAAAACATGTGAATGGCCAAAGTCCCACACATTGTGGACAGATGGAATAGAAGAGA
       ************************************************************

ZIKV   GTGATCTGATCATACCCAAGTCTTTAGCTGGGCCACTCAGCCATCACAATACCAGAGAGG
ZP08   GTGATCTGATCATA
ZP09   GTGATCTGATCATACCCAAGTCTTTAGCTGGGCCACTCAGCCATCACAATACCAGAGAGG
       ************************************************************

ZIKV   GCTACAGGACCCAAATGAAAGGGCCATGGCACAGTGAAGAGCTTGAAATTCGGTTTGAGG
ZP09   GCTACAGGACCCAAATGAAAGGGCCATGGCACAGTGAAGAGCTTGAAATTCGGTTTGAGG
       ************************************************************

ZIKV   AATGCCCAGGCACTAAGGTCCACGTGGAGGAAACATGTGGAACAAGAGGACCATCTCTGA
ZP09   AATGCCCAGGCACTAAGGTCCACGTGGAGGAAACATGTGGAACAAGAGGACCATCTCTGA
       ************************************************************

ZIKV   G
ZP09   G
       *
```

Fig. 1: sequence alignment of ZP08 and ZP09 amplicons with the NS1 region encompassing the 3,805 and 3385 genome position of the Brazilian isolate ZIKV Rio-U1 (KU926309). Star symbol means complete identity with the reference viral sequence in the nucleotide position.

```
ZIKV      AGAGAATCTGGAGTACCGGATAATGCTGTCAGTTCATGGCTCCCAGCACAGTGGGATGAT
ZP17       GAGAATCTGGAGTACCGGATAATGCTATCAGTTCATGGCTCCCAGCACAGTGGGATGAT
          ************************************************************

ZIKV      CGTTAATGACACAGGACATGAAACTGATGAGAATAGAGCGAAGGTTGAGATAACGCCCAA
ZP17      CGTTAATGACACAGGACATGAAACTGATGAGAATAGAGCGAAGGTTGAGATAACGCCCAA
          ************************************************************

ZIKV      TTCACCAAGAGCCGAAGCCACCCTGGGGGGTTTTGGAAGCCTAGGACTTGATTGTGAACC
ZP17      TTCACCAAGAGCCGAAGCCACCCTGGGGGGTTTTGGAAGCCTAGGACTTGATTGTGAACC
          ************************************************************

ZIKV      GAGGACAGGCCTTGACTTTTCAGATTTGTATTACTTGACTATGAATAACAAGCACTGGTT
ZP17      GAGGACAGGCCTTGACTTTTCAGATTTGTATTACTTGACTATGAATAACAAGCACTGGTT
          ************************************************************

ZIKV      GGTTCACAAGGAGTGGTTCCACGACATTCCATTGCCTTGGCACGCTGGGGCAGACACCGG
ZP17      GGTTCACAAGGAGTGGTTCCACGACATTCCATTACCTTGGCACGCTGGGGCAGACACCGG
          ************************************************************

ZIKV      AACTCCACACTGGAACAACAAAGAAGCACTGGTAGAGTTCAAGGACGCACATGCCAAAAG
ZP09                                                                 G
ZP17      AACTCCACACTGGAACAACAAAGAAGCACTGGTAGAGTTCAAGGACGCACATGCCAAAAG
          ************************************************************

ZIKV      GCAAACTGTCGTGGTTCTAGGGAGTCAAGAAGGAGCAGTTCACACGGCCCTTGCTGGAGC
ZP09      GCAAACTGTCGTGGTTCTAGGGAGTCAAGAAGGAGCAGTTCACACGGCCCTTGCTGGAGC
ZP17      GCAAACTGTCGTGGTTCTAGGGAGTCAAGAAGGAGCAGTTCACACGGCCCTTGCTGGAGC
          ************************************************************

ZIKV      TCTGGAGGCTGAGATGGATGGTGCAAAGGGAAGGCTGTCCTCTGGCCACTTGAAATGTCG
ZP09      TCTGGAGGCTGAGATGGATGGTGCAAAGGGAAGGCTGTCCTCTGGCCACTTGAAATGTCG
ZP17      TCTGGAGGCTGAGATGGATGGTGCAAAGGGAAGGCTGTCCTCTGGCCACTTGAAATGTCG
          ************************************************************

ZIKV      CCTGAAAATGGATAAACTTAGATTGAAGGGCGTGTCATACT
ZP09      CCTGAAAATGGATAAACTTAGATTGAAGGGCGTGTCATAC
ZP17      CCTGAAAATGGATAAACTTAGATTGAAGGGCGTGTCATACT
          ****************************************
```

Fig. 2: sequence alignment of ZP09 and ZP17 amplicons with the E region encompassing the 1,373 and 1908 genome position of the Brazilian isolate ZIKV Rio-U1 (KU926309). Star symbol means complete identity with the reference viral sequence in the nucleotide position.
